# Defining a Standard Set of Health Outcomes for Patients With Squamous Cell Carcinoma of the Head and Neck in Spain

**DOI:** 10.3389/fonc.2021.747520

**Published:** 2022-01-24

**Authors:** Virginia Arrazubi, Gerardo Cajaraville, David Cantero, Jordi Giralt, Ricard Mesia, Florencio Monje, Antonio Rueda, Alexander Sistiaga, Jorge Suarez, Alejandro Mut, Marta Comellas, Luis Lizán

**Affiliations:** ^1^ Oncology, Complejo Hospitalario de Navarra, Pamplona, Spain; ^2^ Hospital Pharmacy, Fundación Onkologikoa, San Sebastián, Spain; ^3^ Quality and Innovation, Organización Sanitaria Integrada (Integrated Health Organisation) (OSI) Barrualde Galdakao, Galdakao, Spain; ^4^ Radiation Oncology, Hospital Universitari Vall d´Hebrón, Barcelona, Spain; ^5^ Medical Oncology, Institut Català d’Oncología, Group Badalona Applied Research Group in Oncology (B-ARGO) Group, Institut d'Investigació Germans Trias i Pujol (IGTP), Badalona, Spain; ^6^ Oral and Maxillofacial Surgery, Hospital Universitario de Badajoz, Badajoz, Spain; ^7^ Oncology, Hospital Regional Universitario de Málaga, Málaga, Spain; ^8^ Otolaryngology, Hospital Universitario Donostia, San Sebastián, Spain; ^9^ Bristol Myers Squibb, Madrid, Spain; ^10^ Outcomes’10, Castellón de la Plana, Spain; ^11^ Medicine Department, Jaume I University, Castellón de la Plana, Spain

**Keywords:** head and neck cancer, patient-centered care, outcome measurement, patient-reported outcomes, patient centricity, quality of life

## Abstract

**Purpose:**

A systematic, standardized collection of health outcomes during patient treatment and follow-up, relevant from the perspective of all stakeholders, is a crucial step toward effective and efficient disease management. This project aimed to define a standard set of health outcomes for patients with squamous cell carcinoma of the head and neck (SCCHN).

**Methods:**

The project was led and coordinated by a scientific committee (SC). It comprised: (1) a literature review (to identify variables used during SCCHN management); (2) 1^st^-SC meeting (to select the variables for presentation during nominal groups-NG); (3) five NG (n=42 experts) and four interviews with patients (to reach consensus on the variables for inclusion); and (4) final-SC meeting (to review the results of NG ensuring consensus on the variables where consensus was not reached).

**Results:**

Experts agreed to include the following variables in the standard set: treatment-related (treatment intent and type, response to treatment, treatment toxicity/complication, treatment completion), degree of health (performance status, patient-reported health status, pain, dysphonia, feeding and speech limitations, body image alteration, tracheotomy), survival (overall and progression-free survival, cause of death), nutritional (weight, nutritional intervention), other variables (smoking status, alcohol consumption, patient satisfaction with aftermath care, employment status), and case-mix variables (demographic, tumor-related, clinical and nutritional factors).

**Conclusions:**

This project may pave the way to standardizing the collection of health outcomes in SCCHN and promote the incorporation of patients’ perspective in its management. The information provided through the systematic compilation of this standard set may define strategies to achieve high-quality, patient-centered care.

## 1 Introduction

Head and neck cancer includes a group of neoplasms of various anatomical sites that differ in terms of etiology, diagnostic and treatment approaches (1). It was the seventh most common cancer worldwide in 2020 accounting for 932,000 new cases and 466,500 deaths (2). In Spain, it was estimated that 14,200 new cases of head and neck cancer would be diagnosed in 2020 (3). More than 90% of cases are squamous cell carcinomas of the head and neck (SCCHN) (4) and more than 60% of patients with SCCHN present with stage III or IV disease (5). SCCHN is typically diagnosed in older patients, with smoking and alcohol consumption being two of the main risk factors for its development (1). Moreover, human papilloma virus (HPV) infection has emerged as a new risk factor, especially in oropharyngeal cancer (6).

Treatment for SCCHN is complex and requires a multidisciplinary approach since it differs according to the stage of the disease, anatomical site, and surgical accessibility. It may require intricate surgery, radiation, chemotherapy, and/or targeted therapy, and/or immunotherapy (7, 8). Some of these treatment options involve changes to critical structures for speaking, eating and breathing, which can lead to functionality problems. Therefore, as a result of treatment, patients with SCCHN face long-term challenges beyond surveillance for recurrent or secondary cancer, including adapting to disfigurement, managing dysphagia and developing alternative speech (9, 10).

In addition to curative intention, structural and functional preservation, amelioration of morbidities when feasible, and long-term maintenance of health-related quality of life (HRQoL) are the principal treatment objectives. Therefore, treatment selection based on a multidisciplinary tumor board decision is essential (11, 12). There is growing evidence supporting the routine collection of patient-reported outcomes (PROs) to enable improved, patient-centered care. The systematic collection of PROs in oncology has a positive effect on patient-physician communication, improves the monitoring of disease progression and response to therapy, helps identify unrecognized problems (physical, emotional and/or social problems), contributes to detecting adverse effects of treatment, and enhances patients’ experience and satisfaction (13). Despite the collection of PROs being considered the cornerstone for achieving the best results and preserving patients’ HRQoL, their systematic collection using standardized and validated instruments is mostly limited to clinical research environment, with scarce use in clinical practice.

To move toward an effective and efficient patient-centered system, a holistic approach is required, integrating evidence from clinical outcomes and PROs. Experience gained from other fields shows that the systematic and standardized collection of outcomes is the *sine qua non* to improve the quality of any process (14). During the last few years, pioneer initiatives such as the one performed by the International Consortium for Health Outcomes Measurements (ICHOM) (15) have focused on the development of standard sets of health outcomes for various diseases, among which SCCHN is not included. The long-term goal of these initiatives is to promote consistency in data collection between different institutions within the same country or among different countries. A systematic, standardized compilation of health outcomes, relevant from the perspective of all stakeholders, during patient treatment and follow-up, is a key step toward effective and efficient disease management. This project aimed to define a standard set of health outcomes and the most appropriate instruments to measure them for managing patients diagnosed with SCCHN. This is the first step to ensure to standardize the collection of health outcomes in SCCHN.

## 2 Materials and Methods

**Figure 1:**
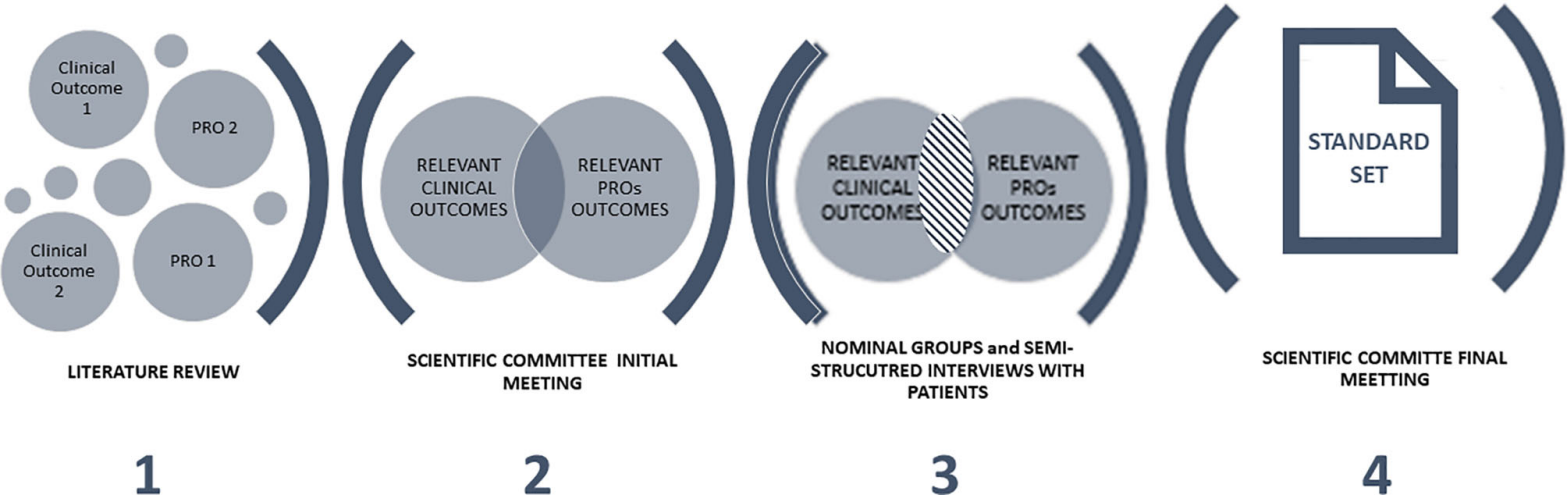
Project phases.

The project comprised four phases: (1) a literature review; (2) first scientific committee meeting; (3) five nominal groups (described in greater detail below) and four semi-structured interviews with patients, and; (4) final scientific committee meeting (**Figure 1**). The scientific committee and nominal group meetings were conducted between June 2019 and December 2020. The project was led and coordinated by a scientific committee consisting of healthcare professionals who are experts in the management of SCCHN, and/or have experience in implementing strategies to standardize health outcomes (three specialists in medical oncology [VA, RM, AR], one specialist in radiation oncology [JG], one specialist in otolaryngology [AS], one specialist in oral and maxillofacial surgery [FM], one specialist in quality and innovation [DC], one hospital pharmacist [GC]) and one representative of a Spanish patient advocacy group (*Grupo Español de Pacientes con Cancer*, GEPAC).

### 2.1 Literature Review

To identify health outcomes [clinical and patient-reported outcomes (PROs)], instruments, and frequency of measurement to be used during SCCHN patient follow-up, a systematic literature review according to the Cochrane Handbook for Systematic Reviews of Interventions (16) (**Supplementary Table S1**), was carried out in Medline/PubMed. Clinical trials or systematic reviews that include clinical trials in SCCHN, published in English and/or Spanish between 01/01/2016 and 03/31/2019 were reviewed.

### 2.2 First Scientific Committee Meeting

The first meeting with the members of the scientific committee aimed to present the project, define the target population and, based on the results of the literature review, select the health outcomes for presenting during the nominal groups.

During the discussion group, the scientific committee screened health outcomes (variable/instrument/frequency of measurement) identified in the literature review and selected them according to their relevance for patient follow-up and availability in the Spanish setting. Moreover, the scientific committee proposed new health outcomes not previously identified in the literature review, but relevant from their perspective.

### 2.3 Nominal Group Meetings and Semi-Structured Interviews With Patient Representatives

Five nominal multidisciplinary groups were conducted to reach a consensus on the health outcomes for inclusion in the standard set. Nominal group meetings took place either face-to-face (n=4 meetings) or online (n=1 meeting), depending on participants’ availability to meet.

A nominal group is a qualitative methodology that allows reaching a consensus and ensuring balanced participation among group members, giving them equal opportunities to share their opinions (17). Following the methodological recommendation (18), nominal groups involved five main steps: 1) Introduction and explanation: welcome and description of the purpose and procedure of the meeting; 2) Silent generation of ideas: each participant individually (without consulting or discussing with others) evaluated the health outcomes proposed; 3) Sharing ideas: separately, participants shared the health outcomes they had selected; 4) Group discussion: participants could seek a verbal explanation or further details about any of the health outcomes that other participants had proposed; 5) Voting and ranking: during this phase, participants were asked to prioritize the health outcomes proposed. Health outcomes were included if ≥ 75% of participants agreed on their inclusion. The five nominal groups worked on the same standard set (based on scientific committee proposal). An individual consensus on specific standard set was reached in each of the nominal group.

The participation in each nominal group was limited to a maximum of 12 experts. Nominal groups consisted of experts from different geographic areas of Spain, including medical oncologists, radiation oncologists, otolaryngologists, oral and maxillofacial surgeons, a phoniatrician, primary care specialists, hospital pharmacists, hospital managers, psycho-oncologists, nutritionists, speech therapists, and dentists. Members of the nominal groups were identified by the scientific committee, in collaboration with the study coordinator. They were selected based on their experience in SCCHN management, PRO measurement, implementing strategies to standardize health outcomes, as well as their availability and interest in the project.

To gain patients’ perspectives on the impact of the disease and its treatment on their day-to-day lives, semi-structured telephone interviews (**Supplementary Table S2**) were conducted with four patient representatives.

### 2.4 Second Scientific Committee Meeting

The main objective of the last meeting of the scientific committee was to define the final standard set for SCCHN. For this purpose, the agreed health outcomes (clinical and PROs) in each nominal group were presented to the scientific committee. The scientific committee reviewed the individual results of the five nominal groups, ensuring consensus on the health outcomes for which no agreement was reached among the nominal groups. Therefore, if a health outcome did not reach the consensus of inclusion in the five nominal groups, the scientific committee members assessed its inclusion or exclusion from the final standard set. The consensus was reached if ≥ 75% of the members of the scientific committee agreed on the inclusion/exclusion of the health outcome.

Additionally, results from semi-structured interviews with patients were also presented to confirm that the most relevant health outcomes from the patients’ perspective were included in the standard set.

Based on the meeting results, the health outcomes for inclusion in the standard set for SCCHN were defined.

## 3 Results

### 3.1 Literature Review

**Figure 2:**
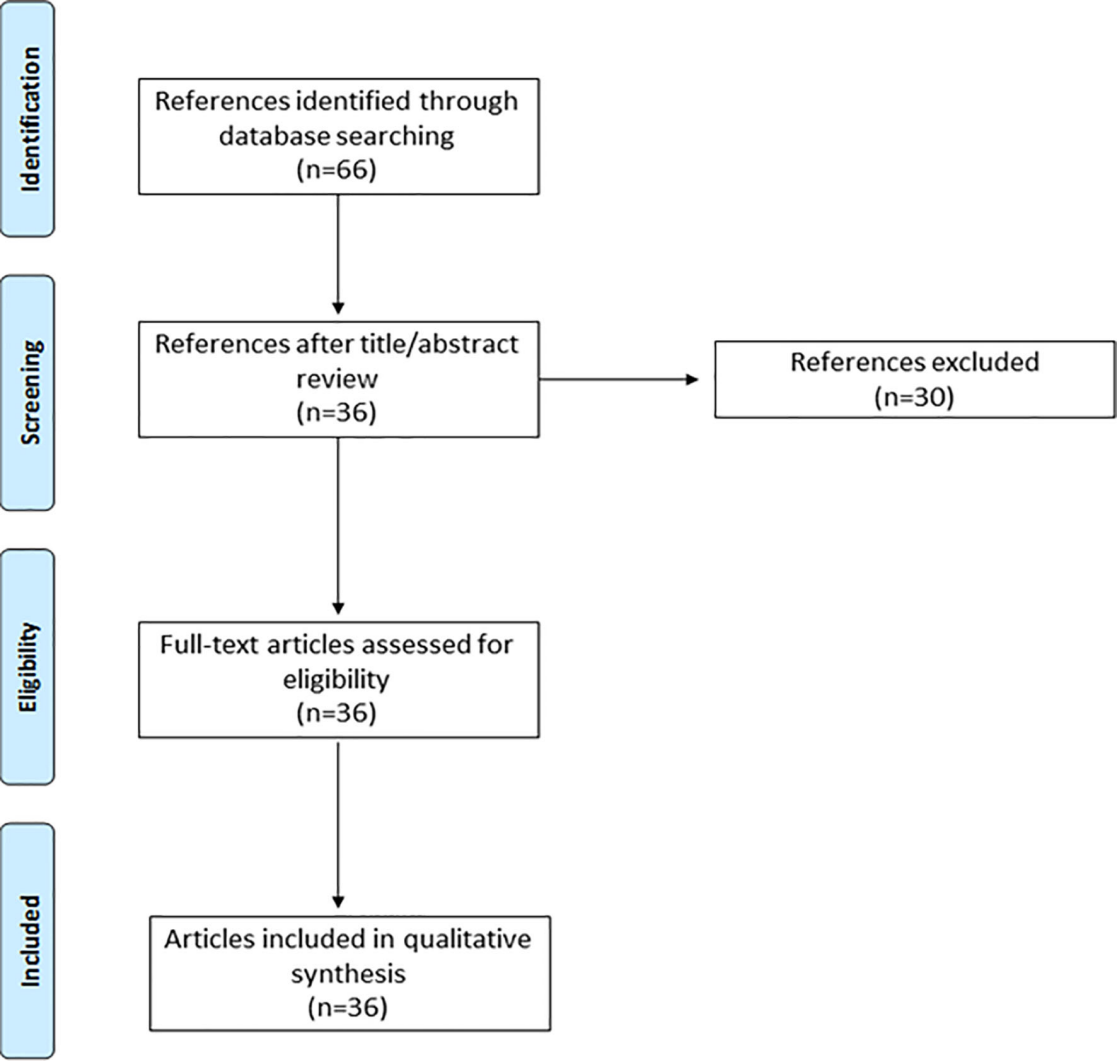
PRISMA flow diagram.

The database search yielded 66 references of which 30 were excluded as their title and abstract contained detailed reviews that did not report health outcomes for patient follow-up. The remaining 36 publications were assessed for eligibility. All of them were selected to be reviewed for qualitative synthesis and identification of health outcomes, measuring instrument, and frequency (**Figure 2**).

A total of 47 health outcomes were identified in the literature review. They were categorized into case-mix variables (baseline factors that may affect the health outcomes but cannot be controlled as part of the management of the condition and enable patient characterization) (n=27) and 20 outcomes variables (variables for patient follow-up) (n=20) (**Supplementary Table S3**).

### 3.2 First Scientific Committee Meeting

The scientific committee considered 18 out of 27 case-mix and 11 out of 20 outcomes variables previously identified in the literature as being relevant. Moreover, two additional case-mix and nine outcomes variables were proposed. Thus, 20 case-mix and 20 outcomes variables were selected by the scientific committee for presentation and evaluation during the nominal groups (**Supplementary Table S4**).

The target population of the standard set was also defined. The scientific committee agreed to include all patients with newly-diagnosed SCCHN originating from the oral cavity, oropharynx, hypopharynx and larynx. Head and neck cancers originating from salivary glands, nasopharynx, paranasal sinuses and nasal cavity, and those with a histological type other than squamous, have special characteristics and are subject to specific recommendations and were therefore not included in the target population of this standard set.

### 3.3 Nominal Group Meetings

A total of 42 experts on SCCHN from different specialties (n=12 medical oncologists, n=4 radiation oncologists, n=6 otolaryngologists, n=2 oral and maxillofacial surgeons, n=1 phoniatrician, n=1 primary care specialist, n=10 hospital pharmacists, n=1 hospital manager, n=2 psycho-oncologists, n=1 nutritionist, n=1 speech therapist, and n=1 dentist) and geographical areas of Spain participated in four nominal group meetings.

The experts agreed to include twelve case-mix and thirteen outcomes variables proposed by the scientific committee in the standard set. Additionally, thirteen new case-mix and thirteen outcomes variables were proposed during the nominal groups (**Supplementary Table S5**).

### 3.4 Semi-Structured Interviews With Patients

Four patient representatives participated in the semi-structured interviews (100% men, age range: 54-82 years).

Patients described the impact of the disease and its treatment on HRQoL, mainly due to disease aftermath. The most common symptoms and side effects reported by patients included dry mouth, oral pain, fatigue and loss of taste and smell. Moreover, several dysfunctions such as speech or voice and swallowing problems were also pointed out.

The disease also harmed patients’ working lives, as most of the patients who were active at the time of diagnosis were unable to return to work.

### 3.5 Second Scientific Committee Meeting

Based on the consensus reached among nominal groups, the scientific committee assessed the inclusion or exclusion of the new health outcomes proposed and those for which the nominal groups did not reach a consensus.

#### 3.5.1 Case-Mix Variables

Case-mix variables are defined as factors that may affect the health outcomes but cannot be controlled as part of the management of the condition. The experts agree to collect at baseline the main sociodemographic factors (age, gender, and social/familiar support), tumor-related factors (tumor localization and sublocalization, cancer staging based on TNM status, and date of diagnosis), clinical factors (alcohol consumption; smoking status, performance status, comorbidities, global patient health status, pain, dysphagia, dysphonia, p16 expression, PD-L1 expression, fragility, and referral to dentistry), and nutritional factors (unintentional weight loss, and dental problem) at the time of diagnosis, before initiating treatment (baseline visits) (**Table 1**).

**Table 1:** Spanish standard set of patient-centered outcomes in SCCHN.

Patient profile	Variable	Supporting information	Measurement instrument	Timing	Data sources
**Sociodemographic factors**							
All patients	Age		Date of birth	Baseline (before treatment begins)	Clinical report
	Gender		F: female; M: male		Clinical report
	Family support	Assessment of patient’s environment/support as a proxy for predicting whether he/she will be able to cope with the disease and complete treatment	(1) Yes; (2) No		Physician-reported
**Baseline tumor factors**							
All patients	TNM status		TNM scale	Baseline (before treatment begins)/after pathological anatomy results (if available)	Clinical report
	Tumor localization and sub localization		NA		Clinical report
	Date of diagnosis		NA		Clinical report
**Baseline clinical factors**							
Patients with oropharyngeal cancer	p16 expression		(1) positive; (2) negative		Clinical report
Recurrent metastatic cancer	PD-L1 expression		(1) positive; (2) negative		Clinical report
Patients >70 years	Fragility		G8 questionnaire		Physician-reported
All patients	Alcohol consumption	Alcohol consumption at diagnosis	(1) Consumer (regular or occasional consumer); (2) Non-consumer	Baseline (before treatment begins)	Physician-reported according to patient-notification
	Smoking status	Smoking status at diagnosis	1) Never-smoker; (2) Ex-smoker (stopped >1 year before diagnosis): years + PYI <10 or PYI ≥10; (3) Current smoker: PYI <10 or PYI ≥10		Physician-reported according to patient-notification
	Performance status		ECOG scale		Physician-reported
	Comorbidities		Comorbidities list*		Clinical report
	Patient-reported health status	Global health and impact of the disease on physical, social and emotional function	Tracked *via* generic questionnaire EQ5D and H&N specific questionnaire EORTC QL-Q H&N43		Patient-reported
	Pain		Tracked *via* items 31,32, 34, 63 of EORTC QL-Q H&N43		Patient-reported
	Referral to dentistry		(1) Yes; (2) No		Physician-reported
	Dysphagia	Swallowing problems	Tracked *via* items 35-38 of EORTC QL-Q H&N43		Patient-reported
	Dysphonia		Tracked *via* items 47, of 55-58 EORTC QL-Q H&N43		Patient-reported
**Baseline nutritional factors**							
All patients	Unintentional weight loss	Unintentional weight loss during the previous 3 months	Yes/No/I don’t know	Baseline (before treatment begins)	Patient-reported
	Dental problems	Loss of teeth or dental problems	Tracked *via* items 39, 40 and 73 of EORTC QL-Q H&N43		Clinical report

PYI, Pack-year index; NA, not applicable; ECOG, Eastern Cooperative Oncology Group; PD-L1, Programmed death-ligand; EQ-5D, EuroQol; EORTC, quality of life core questionnaire; H&N, head and neck cancer; *includes: vasculopathy, renal failure, liver failure, anemia, neuropathy, deafness, diagnosed mental illness, other primary cancer, diabetes, COPD, heart disease, and autoimmune disease. Case-mix variables.

Social/familial support was defined as a proxy for predicting whether the patient would be able to cope with the disease and complete treatment from the physician’s perspective (yes/no).

To standardize the assessment of alcohol consumption, a consensus was reached to classify patients as consumers (regular or occasional) or non-consumers from the patient’s perspective. Similarly, to collect smoking status at diagnosis in a standardized way, the experts agreed to report if the patient was a never-smoker, an ex-smoker (defined as a patient who has stopped at least one year before diagnosis), or a current smoker. For ex-smoker and current smoker patients, it was agreed to record whether the patient’s pack-year index (calculated by multiplying the number of packs of cigarettes smoked per day by the number of years the person has smoked) was < or ≥10.

To systematically report patients’ comorbidities, a list of diseases that can influence treatment and/or clinical practice was developed to select patient comorbidities. The list includes: vasculopathy, renal failure, liver failure, anemia, neuropathy, deafness, diagnosed mental illness, other primary cancer, diabetes, COPD, heart disease, and autoimmune disease.

The experts agreed to use validated questionnaires to assess and collect some of these case-mix variables. To describe patients’ performance status, experts agreed on the use of the Eastern Cooperative Oncology Group (ECOG) scale, which assesses patients’ level of functioning in terms of self-care, carrying out daily activities, and physical ability (19). A generic HRQoL questionnaire (EQ-5D) (20, 21) and head and neck cancer-specific HRQoL questionnaire [EORTC QL-Q H&N43 (22)] were proposed to assess patients’ global health status and the impact of the disease on their social, working and personal function. The use of EORTC QL-Q H&N43 also allows gathering information about patients’ perspective of pain, dysphagia, and dysphonia. And finally, to evaluate patients’ fragility, the experts agreed to complete the G8 questionnaire (23) in patients over 70 years of age.

#### 3.5.2 Outcomes Variables

Outcomes variables establish the evolution of patients’ health status and determine the response (success) in managing the medical condition (**Table 2**). They are collected during patient follow-up.

**Table 2:** Spanish standard set of patient-centered outcomes in SCCHN.

Patient profile	Measure	Supporting information	Measurement instrument	Timing	Data sources
**Treatment variables**					
All patients	Treatment intent		(1) curative; (2) palliative	Baseline (before treatment begins)	Physician-reported
	Type of treatment		(1) Surgery; (2) Radiotherapy; (3) Chemotherapy: (4) Immunotherapy; (5) Targeted therapy; (6) Supportive therapy	Baseline (before treatment begins)	Physician-reported
	Response to curative treatment		(1) Disease-free; (2) Persists; (3) Progress	At 3 months after treatment ends	Clinical report
	Response to palliative treatment	Using RECIST criteria	(1) Complete response; (2) Partial response; (3) Progressive disease; (4) Stable disease	Every 3 cycles of treatment	Clinical report
	Treatment toxicity or surgical complication	Development of treatment toxicity or surgical complications that have interfered with or modified treatment plan	(1) Yes; (2) No	During treatment or surgery	Physician-reported (according to clinical report or patient’s perspective)
	Treatment plan completed		(1) Yes; (2) No; due to lack of efficiency; (3) No, due to toxicity; (4) No, due to patient’s death; (5) No, due to intermittent cause	At treatment end	Physician-reported
**Degree of health**					
All patients	Performance status		ECOG scale	1st year: every 3 m/2nd-3rd years: every 6 m/later: every year	Clinical report
	Patient-reported health status	Global health status, physical and emotional function	Tracked *via* generic questionnaire EQ5D and SCCHN specific questionnaire EORTC QL-Q H&N43	1st year: every 6 m/later: every year	Patient-reported
	Pain		Tracked *via* items 31-34, 63 of EORTC QL-Q H&N43		Patient-reported
	Dysphonia		Tracked *via* items 47, of 55-58 of EORTC QL-Q H&N43		Patient-reported
	Feeding limitations	Includes: dysphagia, dental problems, xerostomia, taste/smell alteration, chewing/eating problems	Tracked *via* items 35-45, 51-54, 73 of EORTC QL-Q H&N43		Patient-reported
	Oral communication limitations	Includes hoarseness and problems talking	Tracked *via* items 47, 55-58 EORTC QL-Q H&N43		Patient-reported
	Body image alteration	Include body image and sexual limitation	Tracked *via* items 48-50, 59-61 of EORTC QL-Q H&N43		Patient-reported
	A requirement for permanent tracheotomy		(1) Yes; (2) No	When tracheotomy is required	Clinical report
**Survival**					
All patients	Overall survival		Date of death	NA	Administrative data (death registry)
	Progression-free survival		NA	1^st^ year: every 3 m/2^nd^-3^rd^ years: every 6 m/later: every year	
	Cause of death	Tumor/treatment-related or not	NA	NA	Administrative data (death registry)
**Nutritional variables**					
All patients	Weight		NA	1st year: every 3 m/2nd-3rd years: every 6 m/later: every year	Clinical report
	Nutritional intervention	Nutritional intervention required during treatment or follow-up	(1) Yes, oral supplementation; (2) Yes, tube enteral nutrition tube; (3) yes, enteral nutrition by ostomy; (4) Not required	When nutritional intervention is required	Clinical report
**Others**					
All patients	Smoking status	Reported if patient still smokes:	(1) Yes; (2) No; (3) Patient did not smoke prior to diagnosis	1st year: every 3 m/2nd-3rd years: every 6 m/later: every year	Physician-reported according to patient-notification
	Alcohol consumption	Reported if patient still consumes alcohol	(1) Yes; (2) No; 3) Patient did not drink alcohol prior to diagnosis	1st year: every 3 m/2nd-3rd years: every 6 m/later: every year	Physician-reported according to patient notification
	Patient satisfaction with aftermath care		(1) Satisfied;(2) Not satisfied; (3) Patient does not have an aftermath	After hospital discharge or 5 years after treatment ends	Patient-reported
	Employment status	Record whether the patient has been able to return to their previous job in the same conditions	(1) Yes;(2) No; (3) Patient did not work prior to diagnosis	After oncology department discharge or 5 years after treatment ends	Patient-reported

NA, not applicable; ECOG, Eastern Cooperative Oncology Group; EQ-5D: EuroQol; EORTC, quality of life core questionnaire; H&N: head and neck cancer. Outcomes variables.

##### 3.5.2.1 Treatment-Related Variables

It was agreed to collect, prior to initiating treatment, data on treatment intention and type. For patients that receive curative treatment, the experts reached a consensus on the inclusion of treatment response at three months after end of treatment. For patients receiving palliative treatment, it was agreed to assess response to treatment using RECIST criteria every three cycles of treatment.

The experts agreed to report if the patient had developed treatment toxicity or any surgical complication interfering with or modifying the treatment plan. Finally, the experts considered it relevant to indicate if the patient had completed the treatment plan and, when applicable, record the reason for not doing so.

##### 3.5.2.2 Degree of Health

SCCHN has a negative impact on patients’ performance and health status. Therefore, it was agreed to collect both sets of variables during patient follow-up. As previously indicated, the ECOG scale (19) was selected to assess patients’ performance status, while the EQ-5D (20, 21) and EORTC QL-Q H&N43 (22) were chosen for the assessment of patients’ HRQoL. The use of EORTC QL-Q H&N43 annually during patient follow-up allows the evaluation of the impact of the disease on patients’ lives, including pain, dysphonia, feeding limitations, oral communication limitations, and body image alterations.

Given the significant impact that it can have on patients, it was also agreed to collect information on whether or not the patient requires a permanent tracheotomy.

##### 3.5.2.3 Survival

Overall survival and progression-free survival were considered key variables for inclusion in the standard set for patient follow-up. Moreover, participants agreed on gathering information regarding cause of death, indicating whether it was tumor- or treatment-related.

##### 3.5.2.4 Nutritional Variables

The disease and its treatment have a negative impact on patients’ nutritional status. For this reason, it was agreed to collect patients’ weight at each visit, and record whether the patient had required nutritional intervention during treatment or follow-up.

It is important to note that the use of EORTC QL-Q H&N43 allows the physician to assess dysphagia, dental problems, xerostomia, taste/smell alterations, and chewing/eating problems that may impact patients’ nutritional status.

##### 3.5.2.5 Others

Alcohol and tobacco consumption are the main risk factors for SCCHN development, and their maintenance during and/or after treatment is related with recurrence, second neoplasms and tobacco/alcohol-related death. Consequently, it was agreed to record whether the patient continued smoking or consuming alcohol after diagnosis.

SCCHN may also affect patients’ employment status; therefore, the experts agreed to report whether patients can return to their previous job under similar conditions after discharge from the oncology department or five years after end of treatment.

Most patients with SCCHN reported difficulty in access to aftermath care. Therefore, it was agreed to record whether patients were satisfied with the aftermath care received after being discharged from the oncology department and five years after treatment end.

## 4 Discussion

A systematic and standardized collection of health outcomes during follow-up of patients with SCCHN is a crucial step toward a more effective and efficient healthcare system. A holistic approach, integrating all stakeholders’ perspectives, is necessary to ensure the best quality care. To this end, a standard set that includes relevant health outcomes from the perspective of both patients and healthcare professionals is required. The SCCHN standard set defined herein is an excellent opportunity to promote patient-centered care and optimize SCCHN management.

The SCCHN standard set includes 21 outcomes variables. In addition to traditional variables regarding survival or treatment, eight are included related to patients’ degree of health (performance status, patient-reported health status, pain, dysphonia, feeding limitations, oral communication limitations, body image alteration, and need for permanent tracheostomy). Six of them are proposed for tracking *via* HRQoL questionnaires, EQ-5D-3L and EORTC QL-Q H&N43. SCCHN and its treatment can compromise vital functions, such as breathing, swallowing, and speech. Therefore, the disease can lead to significant physical, emotional, and social problems, reducing patients’ HRQL. Although the collection of HRQoL and other PROs is scarce in clinical practice, the inclusion of these variables in the standard set was considered key to establishing the impact of the disease from the patients’ perspective (24, 25). Due to the lack of resources and the limited knowledge of these questionnaires in clinical practice (26), it was proposed to complete these questionnaires every six months during the first year and then annually. Moreover, by using the EORTC QL-Q H&N43 questionnaire, information about the impact of the disease and its treatment on nutritional status, such as dysphagia, dental problems, dry mouth, sensory problems, taste/smell alterations, problems with chewing, and weight loss, can also be gathered.

Although some healthcare professionals perceive the use of PROs in clinical practice as time-consuming, a one-year pilot study conducted in the Netherlands demonstrated that the ICHOM standard set could be implemented during routine SCCHN treatment without significantly disturbing the everyday workflow (27, 28). The authors concluded that the collection of PROs is not overly time-consuming; however, it requires *ad hoc* tools and dedicated staff (27, 28).

Providing patient-centered care is essential to move toward high-quality integrated care. Therefore, the inclusion of PROs in the standard set is crucial. Other initiatives have been conducted to promote the use of PROs and patient-reported experiences (PREs) to measure the quality of care, showing that PROs and PREs are promising for measuring and improving the quality and personalization of healthcare in patients with SCCHN (29, 30).

In addition to defining the essential variables for patient follow-up, the experts agreed on those necessary to characterize the patient, the case-mix. The inclusion of these variables is beneficial for benchmarking purposes and for comparing results based on patient profiles.

Data from the implementation of other standard sets in clinical practice has shown benefits from patients’ and clinicians’ perspectives. On the one hand, the inclusion of PROs in the standard set will allow clinicians to focus on the aspects of the disease that most matter to the patient, and therefore encourage better patient engagement in disease management. Moreover, clinicians can learn from the outcomes data they gather, and from the experience of other healthcare professionals in different settings, the standard set thus becoming a valuable tool for benchmarking (31–34).

This project presents several limitations. This standard set reflects the opinion of a group of 50 experts on the management of SCCHN, four patient representatives (all men) and one patient advocacy group representative. Although no significant differences are expected, different groups of experts and patients, including women, could have agreed on various other recommendations. To minimize this potential bias and ensure national representativeness, participants from four broad geographic areas were involved in the project. Secondly, some health outcomes, for instead data regarding surgical details are finally excluded from the standard set. In this regard, it is important to bear in mind that we aim to achieve a minimum, standard set to ensure that at a minimum these health outcomes are collected. In third place, some relevant variables, such as biomarkers may not have been considered when elaborating on the standard set. To minimize this limitation, and due to the continuing advances in both the knowledge and treatment of this disease, we recommend periodically updating the list of biomarkers for evaluation during patient follow-up. We also suggest that the present standard set be regularly updated.

Although this standard set for SCCHN marks a starting point, several barriers need to be overcome on the road to its successful implementation in the Spanish setting. Namely, the time required for the collection of the health outcomes proposed, the lack of digital tools allowing systematic and automatic PRO measurements (PROMs) compilation, together with limited education and information of patients and clinicians about PROs, have been identified as the main barriers to the implementation of the present standard set (35). Newer platforms for data collection, based on information and communication technologies, may reduce the burden on both patient and clinician, as well as data processing time, thus facilitating the use of PROs in clinical practice (36). It is important to notice that, regardless of the platform used to collect the health outcomes of the standard set, they will be included in the patient’s medical record; therefore, the protection of personal data will be guaranteed and will follow the same procedure as the rest of the data in the medical record. Other barriers that must be overcome to ensure the widespread use of this standard set are inherent to the structure of the Spanish national healthcare system (SNHS). Indeed, one of the main characteristics of the SNHS is its heterogeneity: healthcare processes, organizational models as well as information systems differ widely both among and within regions.

Besides addressing these barriers, a further step to promote the integration of the defined standard set into the Spanish healthcare model may involve conducting a pilot implementation study. A pilot study may help establish the feasibility of introducing the standard set in the routine clinical practice, providing insights into the leading resource requirements and organizational challenges to be tackled during implementation.

## 5 Conclusion

The standard set defined may pave the way to standardizing the collection of variables in SCCHN and contribute to promoting the incorporation of patient perspective in SCCHN management. In turn, the information provided through the systematic compilation of this set of health outcomes may allow both clinicians and health policymakers to define strategies aimed at achieving high-quality, patient-centered care.

## Data Availability Statement

The original contributions presented in the study are included in the article/**Supplementary Material**. Further inquiries can be directed to the corresponding author.

## Author Contributions

VA, GC, DC, JG, RM, FM, AR, and AS: contributed equally to this work with data acquisition and data interpretation. JS, AM, and LL contributed to the conceptualization and design of the study. JS and AM did not participate either as members of Scientific Committee or on the selection of members of the Scientific Committee. MC contributed to the conceptualization and design of the study, data acquisition, data analysis, data interpretation and wrote the first draft of the manuscript. All authors contributed to manuscript revision, read and approved the submitted version.

## Funding

The project was sponsored by Bristol Myers Squibb. The funder was not involved in the study design, collection, analysis, interpretation of data, the writing of this article or the decision to submit it for publication.

## Conflict of Interest

JS and AM are employees of Bristol Myers Squibb. MC and LL work for an independent research entity that received funding from Bristol Myers Squibb to coordinate and conduct the study.

The remaining authors declare that the research was conducted in the absence of any commercial or financial relationships that could be construed as a potential conflict of interest.

## Publisher’s Note

All claims expressed in this article are solely those of the authors and do not necessarily represent those of their affiliated organizations, or those of the publisher, the editors and the reviewers. Any product that may be evaluated in this article, or claim that may be made by its manufacturer, is not guaranteed or endorsed by the publisher.
