# Supplementary material for: Defining a Standard Set of Health Outcomes for Patients With Squamous Cell Carcinoma of the Head and Neck in Spain
Source: Front Oncol. 2022 Jan 24;11:747520. doi: 10.3389/fonc.2021.747520 (PMC8819151; doi:10.3389/fonc.2021.747520)
Supplement: Supplementary file 1 [file Table_1.docx]

Supplementary Table S1: Search terms and strategy in Medline/Pubmed

| Database | Pubmed/Medline |
| --- | --- |
| **Search period** | January, 1st 2016- March, 31st 2019 (beginning of the project) |
| **Search strategy** | "Squamous Cell Carcinoma of Head and Neck"[Mesh] AND (randomized controlled trial[All Fields] OR randomized controlled trials[All Fields] OR randomized controlled trial[All Fields] OR randomised controlled trials[All Fields] OR randomized controlled trial[Publication Type] OR Review[ptyp] OR systematic[sb]) AND ("Quality of Life"[Mesh] OR "Quality Indicators, Health Care"[Mesh] OR "Patient Outcome Assessment"[Mesh] OR patient reported outcome*[tiab] OR patient related outcome*[tiab] OR patient-reported outcome*[tiab] OR patient-related outcome*[tiab] OR patient reported outcome*[ot] OR patient related outcome*[ot] OR patient-reported outcome*[ot] OR patient-related outcome*[ot] OR "Treatment Outcome"[Mesh]) |
