# Supplementary material for: Defining a Standard Set of Health Outcomes for Patients With Squamous Cell Carcinoma of the Head and Neck in Spain
Source: Front Oncol. 2022 Jan 24;11:747520. doi: 10.3389/fonc.2021.747520 (PMC8819151; doi:10.3389/fonc.2021.747520)
Supplement: Supplementary file 2 [file Table_2.docx]

**Supplementary Table S2. Semi-structured telephone interviews with patients representatives.**

| **Socio-demographic characteristics** | age, sex, diagnosis, years from diagnosis, treatment received (surgery, radiation, chemotherapy, targeted therapy, immunotherapy), current health status |
| --- | --- |
| **Burden of the disease on** | social, working, and personal life |
| **Disease aftermath** | swallowing problems, eating problems, problems eating in public, speech problems, oral cavity problems (pain/discomfort), dental problems, sensory problems (smell/taste), sexual problems, dry mouth, hoarse voice, cough, weight alterations, need for nutritional support, arm mobility, skin alterations, tingling, pain |
| **Most relevant adverse events of treatment** |  |
| **Healthcare professionals involved in patient’s monitoring** |  |
