# Supplementary material for: Defining a Standard Set of Health Outcomes for Patients With Squamous Cell Carcinoma of the Head and Neck in Spain
Source: Front Oncol. 2022 Jan 24;11:747520. doi: 10.3389/fonc.2021.747520 (PMC8819151; doi:10.3389/fonc.2021.747520)
Supplement: Supplementary file 3 [file Table_3.docx]

Supplementary Table S3: Case-mix and outcomes variables identified in the literature

|  | **Variable** | **Num of articles identified** | **Measuring instruments** |
| --- | --- | --- | --- |
| **Case-mix variables** | | | |
| **Sociodemographic factors** | Age | 36 | NA |
|  | Gender | 36 | F: female; M: male |
|  | Place of residence | 1 | Major city / Inner regional /Outer regional |
|  | Employment status | 1 |  |
|  | Educational level | 3 | Secondary or lower / Post-secondary |
|  | Marital/family status | 3 | Marital status: married or widowed / not married  Family Status: lives alone (yes/no) |
|  | Smoking status | 17 | - Pack-year index - never-smoker / ex-smoker / current smoker - smoked/chewed |
|  | Alcohol consumption | 4 | - Consumer / non-consumer - Units - Low risk / Risky |
|  | Income (annual household) | 1 | $0–15,599 / $15,600–36,399 / $35,400–78,000 / $78,001 or more |
| **Tumor related factors** | Tumor localization | 20 | NA |
|  | TNM status | 18 | NA |
|  | Clinical stage | 15 | 0 / I / II / III / IV |
|  | Recurrence/Metastasis | 3 | NA |
|  | Histology | 1 | Well / Moderately / Poorly differentiated / Cannot be assessed / Unknow |
| **Baseline clinical factors** | Status HPV | 6 | Immunohistochemistry and/or PCR |
|  | Status p16 | 7 | Immunohistochemistry |
|  | Malignant lesion | 2 | NA |
|  | Performance status | 15 | - ECOG - Karnofsky Performance Status Scale |
|  | Molecular targets | 2 | EGFR(35), HER3, PTEN, c-Met, TP53, NOTCH1, KDR |
|  | Psychological illnesses | 1 | NA |
|  | Anemic | 1 | NA |
|  | Swallowing problems or dysphagia** | 1 | NA |
| **Nutritional factors** | Weight loss | 5 | - During the last 6 months (kg) - During the last 3 months (kg) |
|  | BMI | 1 | NA |
|  | Nutritional status | 2 | - SGA - PG-SGA |
|  | Muscular mass | 1 | NA |
|  | Type of diet | 1 | Standard/soft/minced and moist/smooth pureed |
| **Outcomes variables** | | | |
| **Survival** | Overall survival | 24 | NA |
|  | Progression-free survival | 18 | NA |
|  | Death related to disease∫ | 4 | NA |
|  | Death not related to disease∫ | 3 | NA |
| **Treatment factors** | Type of treatment | 17 | NA |
|  | Response to treatment | 17 | RECIST |
|  | Adverse events (grade >3) | 11 | CTCAE |
|  | Adherence | 4 | Morisky-Green |
|  | Spinal Accessory Nerve Function~ | 1 | NA |
|  | Perioperative Complications~ | 1 | hematoma / seroma |
|  | Shoulder function~ | 1 | Shoulder abduction / flexion |
| **Degree of health** | Performance status | 3 | - ECOG - Karnofsky Performance Status Scale |
|  | HRQoL | 15 | - generic questionnaire: EQ-5D; - Specific oncologic questionnaire: EORTC QLQ-C30, FACT-G - Specific H&N cancer questionnaire: EORTC QLQ-H&N35, FACT-H&N |
|  | Mood state | 1 | POMS-SF |
|  | Pain | 1 | NRS |
| **Nutritional factors** | Nutritional status | 1 | - SGA - PG-SGA |
|  | Weight | 3 | NA |
|  | % fat | 1 | NA |
|  | Body composition | 1 | Bioelectrical impedance /Lean and fat body mass |

NA, Not applicable; HPV, Human Papilloma Virus; ECOG, Eastern Cooperative Oncology Group; EGFR, epidermal growth factor receptor; HER3, human epidermal growth factor 3; PTEN, phosphatidylinositol-3,4,5-triphosphatase 3-phosphatase; KDR, Kinase insert domain receptor; BMI, body mass index; SG,: subjective global assessment; PG-SGA, patient-generated subjective; RECIST, response evaluation criteria in solid tumors; CTCAE, common terminology criteria for adverse events; HRQoL, health-related quality of life; EORTC, quality of life core questionnaire; FACT, functional assessment of cancer therapy; H&N: head and neck cancer; POMS-SF, short form of the profile of mood states; NRS, numeric rating scale
