# Supplementary material for: Defining a Standard Set of Health Outcomes for Patients With Squamous Cell Carcinoma of the Head and Neck in Spain
Source: Front Oncol. 2022 Jan 24;11:747520. doi: 10.3389/fonc.2021.747520 (PMC8819151; doi:10.3389/fonc.2021.747520)
Supplement: Supplementary file 4 [file Table_4.docx]

Supplementary Table S4: Case-mix and outcomes variables presented and evaluated during the nominal groups.

|  | **Variable** | **Measuring instruments** |
| --- | --- | --- |
| **Case-mix variables** | | |
| **Sociodemographic factors** | Age | NA |
|  | Gender | (1) F: female; (2) M: male |
|  | Employment status | (1) Active worker; (2) Unemployed; (3) Retired or incapacitated due to disease |
|  | Educational level | (1) Primary; (2) Secondary; (3) Post-secondary |
|  | Smoking status | (1) Never-smoker; (2) Ex-smoker (≥1year): years + pack-year; (3) Current smoker: pack-year <10 or pack year >10 |
|  | Alcohol consumption | (1) Consumer (harmful consumer or regular/occasional consumer); (2) Non-consumer |
|  | Family support | (1) Lives with family and has identified caregiver; (2) Lives alone, but has a caregiver identified; (3) Lives alone, without a caregiver |
| **Tumor related factors** | Tumor localization and sub localization | NA |
|  | TNM status | NA |
|  | Clinical stage | (1) 0; (2) I; (3) II; (4) III; (5) IV |
|  | Histology | (1) Well; (2) Moderately; (3) Poorly differentiated; (4) Unknown |
| **Baseline clinical factors** | Status p16 | (1) p16 + (HPV + / HPV - / unknown); (2) p16 - |
|  | Performance status | ECOG |
|  | Molecular targets | PD-L1 |
|  | Comorbidities | Vasculopathy / Kidney failure / Liver failure / Anemia (hemoglobin) / Neuropathy / Deafness / Diagnosed mental illness / Other primary cancer |
|  | Frailty | - G8 questionnaire - Age / comorbidity / Family support |
| **Nutritional factors** | Weight loss | During the last 3 months (kg) |
|  | BMI | NA |
|  | Swallowing problems or dysphagia | Self-reported |
|  | Nutritional status | NutriScore questionnaire |
| **Outcomes Variables** | | |
| **Survival** | Overall survival | NA |
|  | Progression-free survival | NA |
|  | Cause of death | (1) Not related to the disease (treatment or comorbidity); (2) related to the disease |
|  | Event-free survival | Event = Recidivism / 2nd tobacco-related neoplasm / death |
| **Treatment factors** | Type of treatment* | (1) Surgery; (2) Radiotherapy; (3) Chemotherapy: (4) Immunotherapy; (5) Targeted therapy; (6) Supportive therapy |
|  | Response to treatment | Curative treatment: (1) Disease-free; (2) Persists; (3) Progress |
|  |  | Palliative treatment: RECIST (1) Complete response; (2) Partial response; (3) Progressive disease; (4) Stable disease |
|  | Adverse events (grade >3) | Self-reported |
|  |  | CTCAE |
|  | Surgery complications | NA |
|  |  |  |
|  | Treatment intent | (1) curative; (2) palliative |
|  | Treatment completed | (1) Yes; (2) No; due to lack of efficiency; (3) No, due to toxicity; (4) No, due to patient’s death |
| **Degree of health** | Performance status | ECOG |
|  | HRQoL | - generic questionnaire: EQ-5D; - Specific oncologic questionnaire: EORTC QLQ-C30, FACT-G - Specific H&N cancer questionnaire: EORTC QLQ-H&N35, FACT-H&N |
|  | Pain | NRS |
|  | Patient’s aftermath | (1) Yes; Limit: Social/working/personal life; (2) No |
|  | Impact on body image | Body Image Scale |
| **Nutritional factors** | Nutritional status | NutriScore |
|  | Weight | NA |
|  | Nutritional intervention | (1) Oral nutrition; (2) Enteral nutrition: tube/ostomy |
| **Others** | Smoking status | The patient still smokes: (1) Yes; (2) No |
|  | Employment status | (1) Active worker; (2) Unemployed; (3) Retired or incapacitated due to disease |

* Allows combinations between the options

NA, Not applicable; HPV, Human Papilloma Virus; ECOG, Eastern Cooperative Oncology Group; PD-L1, Programmed Death-ligand; BMI, body mass index; RECIST, response evaluation criteria in solid tumors; CTCAE, common terminology criteria for adverse events; HRQoL, health-related quality of life; EORTC, quality of life core questionnaire; FACT, functional assessment of cancer therapy; H&N: head and neck cancer; NRS, numeric rating scale
