# Supplementary material for: Defining a Standard Set of Health Outcomes for Patients With Squamous Cell Carcinoma of the Head and Neck in Spain
Source: Front Oncol. 2022 Jan 24;11:747520. doi: 10.3389/fonc.2021.747520 (PMC8819151; doi:10.3389/fonc.2021.747520)
Supplement: Supplementary file 5 [file Table_5.docx]

Supplementary Table S5: Case-mix and outcomes variables agreed and additionally proposed during nominal group

| **Variable** | | **Nominal group agreed to include (n/N)** |
| --- | --- | --- |
| **Case-mix variable** | | |
| **Sociodemographic factors** | Age | 5/5 |
|  | Gender | 5/5 |
|  | Employment status | 2/5 |
|  | Educational level | 1/5 |
|  | Smoking status | 5/5 |
|  | Alcohol consumption | 5/5 |
|  | Family support / social-familiar support* | 5/5 |
|  | Residence area* | 1/5 |
|  | Passive exposure to risk substances* | 1/5 |
|  | Oral hygiene habits* | 1/5 |
| **Tumor related factors** | Tumor localization and sub localization | 5/5 |
|  | TNM status | 5/5 |
|  | Clinical stage | 2/5 |
|  | Histology | 1/5 |
|  | Date of diagnosis* | 1/5 |
|  | Perineural or vascular invasion* | 1/5 |
| **Baseline clinical factors** | Status p16 | 5/5 |
|  | Performance status | 5/5 |
|  | Molecular target (PD-L1) | 1/5 |
|  | Comorbidities | 5/5 |
|  | Frailty | 2/5 |
|  | HRQoL* | 2/5 |
|  | Previous treatments that may impact surgery* | 1/5 |
|  | Dysphagia* | 1/5 |
|  | Dyspnea* | 1/5 |
|  | Dysphonia* | 1/5 |
|  | Pain* | 1/5 |
| **Nutritional factors** | Weight loss | 5/5 |
|  | BMI | 4/5 |
|  | Swallowing problems or dysphagia | 5/5 |
|  | Nutritional status | 2/5 |
|  | Dental Assessment* | 3/5 |
|  | Odinophagy* | 1/5 |
| **Outcomes Variables** | | |
| **Survival** | Overall survival | 5/5 |
|  | Progression-free survival | 5/5 |
|  | Cause of death | 5/5 |
|  | Event-free survival | 2/5 |
|  | Locoregional recurrence-free survival* | 1/5 |
|  | Disease-free survival* | 1/5 |
| **Treatment** | Type of treatment | 5/5 |
|  | Response to treatment | 5/5 |
|  | Adverse events (grade >3) | 5/5 |
|  | Surgery complications | 2/5 |
|  | Treatment intent | 5/5 |
|  | Treatment completed | 5/5 |
|  | Treatment start and end date* | 1/5 |
| **Degree of health** | Performance status | 5/5 |
|  | HRQoL | 4/5 |
|  | Pain | 3/5 |
|  | Patient’s aftermath | 5/5 |
|  | Impact on body image | 2/5 |
| **Nutritional** | Nutritional status | 3/5 |
|  | Weight | 4/5 |
|  | Nutritional intervention | 5/5 |
|  | Type of diet* | 1/5 |
|  | Dental assessment* | 1/5 |
|  | Dysphagia* | 1/5 |
|  | Odinophagy* | 1/5 |
|  | Trismus* | 1/5 |
|  | Dysgeusia* | 1/5 |
| **Others** | Smoking status | 5/5 |
|  | Employment status | 1/5 |
|  | Alcohol consumption* | 5/5 |
|  | Family Support* | 1/5 |
|  | Access to aftermath care* | 1/5 |
|  | Functional treatments* | 1/5 |
|  | Oral health* | 1/5 |

Highlighted in gray are variables that reached consensus for inclusion among nominal groups; *New variables proposed during nominal group meetings; BMI, body mass index; HRQoL, health-related quality of life.
